# Water–Energy Nexus: Membrane Engineering Towards a Sustainable Development

**DOI:** 10.3390/membranes15040098

**Published:** 2025-03-26

**Authors:** Alessandra Criscuoli

**Affiliations:** Institute on Membrane Technology (CNR-ITM), Via P. Bucci 17/C, 87036 Rende, Italy; a.criscuoli@itm.cnr.it; Tel.: +39-0984-492118

**Keywords:** water–energy nexus, desalination, reverse osmosis, membrane distillation

## Abstract

Sustainable development is linked to the achievement of several different objectives, as outlined by the 17 Sustainable Development Goals (SDGs) defined by the United Nations. Among them are the production of clean water and the combat of climate change, which is strictly linked to the use of fossil fuels as a primary energy source and their related CO_2_ emissions. Water and energy are strongly interconnected. For instance, when processing water, energy is needed to pump, treat, heat/cool, and deliver water. Membrane operations for water treatment/desalination contribute to the recovery of purified/fresh water and reducing the environmental impact of waste streams. However, to be sustainable, water recovery must not be energy intensive. In this respect, this contribution aims to illustrate the state of the art and perspectives in desalination by reverse osmosis (RO), discussing the various approaches looking to improve the energy efficiency of this process. In particular, the coupling of RO with other membrane operations, like pressure-retarded osmosis (PRO), reverse electrodialysis (RED), and forward osmosis (FO), as well as the osmotic-assisted reverse osmosis (OARO) system, are reported. Moreover, the possibility of coupling a membrane distillation (MD) unit to an RO one to increase the overall freshwater recovery factor and reduce the brine volumes that are disposed is also discussed. Specific emphasis is placed on the strategies being applied to reduce the MD thermal energy demand, so as to couple the production of the blue gold with the fight against climate change.

## 1. Introduction

The ambition to pursue sustainable development has attracted scientists for a long time. One of the first definitions, in fact, dates back to the 80s: *“Sustainable Development is a process of change in which the exploitation of resources, the direction of investments, the orientation of technical development, and institutional change are all in harmony and enhance both current and future potential to meet human needs and aspirations”* [1]. However, forty years later, there is still a long way to go to reach sustainability, although extensive research has been conducted and many attempts have been made. The long road to sustainability is due to the fact that sustainable development is obtained at the intersection of three main domains: economy, environment and society. This means that different aspects must be fulfilled at the same time. In this respect, three specific indicators were defined to assess the sustainability of a process: 1. economic indicators, linked to investment costs and profits; 2. environmental indicators taking into account the use of resources and the emissions/pollution produced; 3. social indicators covering the health and safety of employers and the benefits and issues for the community. The United Nations defined the Sustainable Development Goals (SDGs) for the 2030 Agenda as 17 key objectives to be pursued in the future [2].

Among them, the SDG 6 “Clean water and sanitation” aims to ensure the availability of water and sanitation worldwide, while the SDG 13 “Climate action” focuses on actions against climate change.

**Figure 1:**
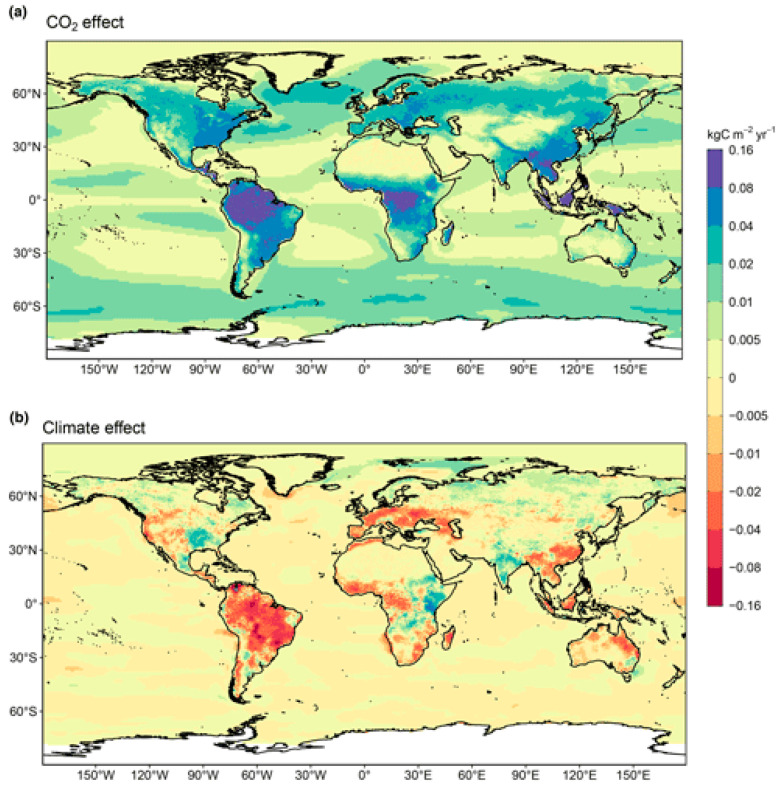
(**a**) Distribution of the CO_2_ concentration in the atmosphere and (**b**) change in climate in 2013–2022. Reproduced from [4] with Open Access.

The availability of water is of crucial importance. In fact, water is life! Water is a major component of the human body, it is used in daily life for hygiene, it is needed by animals and plants, and it is employed in industry. Although about 70% of Earth’s surface is made of water, only 3% is fresh water, of which 2/3 is entrapped into glaciers and 1/3 is distributed between groundwaters and surface waters. Population growth, industrial and agricultural activities, water pollution, and drought exacerbated by climate change significantly impact freshwater availability, causing a continuous increase in water stress. As highlighted by the Sustainable Development Goals Report of the United Nations [3], 2.4 billion people live in water-stressed countries, with a lack of safe management of drinking water and sanitation. Therefore, it requests an accelerated implementation of integrated water resource management. On the other hand, from a world energy outlook, emissions from fossil fuels increased by 1.1% in 2023 compared to 2022, leading to 36.8 billion metric tons of CO_2_ emissions in 2023 [4]. The distribution of the CO_2_ concentration in the atmosphere and the change in climate for the period 2013–2022 are reported in Figure 1a and Figure 1b, respectively.

**Figure 2:**
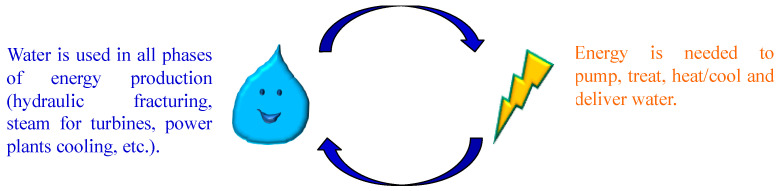
The water–energy nexus.

It is expected that the global warming caused by CO_2_ emissions will further increase drought on the planet, with a consequent impact on water stress. Given this, it appears that the recourse to renewable energies, like solar, wind, geothermal, bioenergy (biofuels) and green hydrogen, is an urgent need. Water and energy are directly linked in the so-called “water–energy nexus”. In fact, water is used in all phases of energy production (e.g., hydraulic fracturing, steam for turbines, cooling and cleaning steps, etc.), while energy is employed to pump, treat, heat, cool, and deliver water (see Figure 2). Today, the sustainable management of water and energy represents a big challenge given the growing water and energy demand, the growing population, and climate change. The treatment of contaminated water, as well as seawater desalination, are important strategies to recover purified/fresh water, thus increasing water availability. In this respect, membrane operations have successfully been employed. Compared to conventional operations, they do not need chemicals to undertake separation and do not involve moving parts (safer). Moreover, membrane processes are characterized by high capacity/size ratio (high compactness), high flexibility and modularity, and easy scale-up. They also present lower energy consumption than conventional systems. However, with the goal of reaching the sustainable management of water and energy, a reduction in the energy consumption of membrane operations is becoming a target of high interest. In this contribution, the efforts made to reduce the energy consumption linked to water treatment by membrane operations are reported. In particular, the latest trends in desalination by reverse osmosis and membrane distillation are presented and discussed, with the aim of identifying the future actions needed to further reduce the energy demand and address the research in the field. Specifically, in addition to the integration of RO with other membrane units to decrease the RO energy consumption, the different strategies in progress to reduce the thermal energy demand of MD working on the RO brine are also analyzed.

## 2. Membrane Operations for Water Treatment

**Figure 3:**
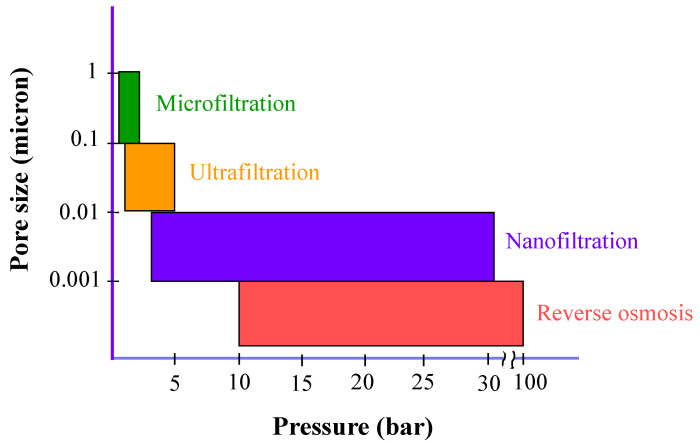
Typical pore sizes and applied pressure of main pressure-driven membrane operations. For the sake of figure readability, the pore size values on the *y*-axis are not to scale.

Membrane operations for water treatment can be divided into two main typologies: pressure-driven and thermal-driven ones. In pressure-driven membrane operations, the driving force is a difference in pressure across the two sides of the membrane, created by pressurizing the water stream at one side of the membrane while keeping the other side at atmospheric pressure. The membranes used are hydrophilic. The smaller the membrane pore size, the higher the pressure that must be applied to allow the permeation of water through the membrane and, consequently, the higher the electric energy consumption. Microfiltration (MF), ultrafiltration (UF), nanofiltration (NF), and RO belong to this typology (see Figure 3), with MF and RO characterized by the highest and lowest electric energy consumption, respectively.

**Figure 4:**
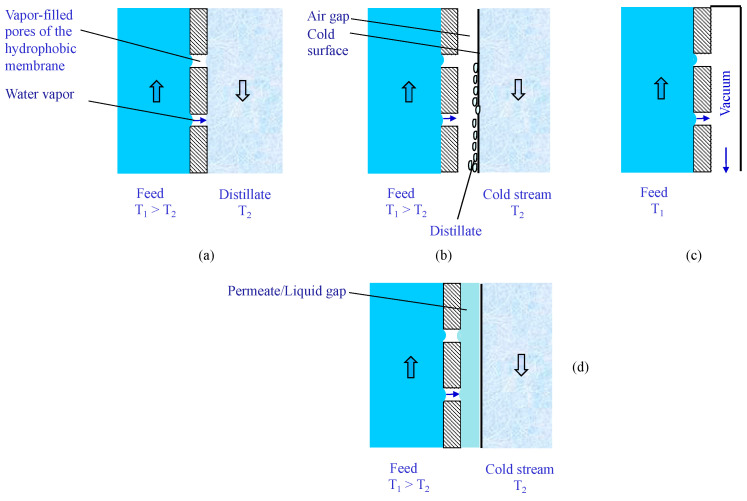
Main MD configurations. (**a**) Direct contact membrane distillation. (**b**) Air gap membrane distillation. (**c**) Vacuum membrane distillation. (**d**) Permeate/liquid gap membrane distillation.

Thermal-driven membrane operations (e.g., membrane distillation) use differences in vapor pressure across the membrane as their driving force. In this case, membranes are hydrophobic with a pore size in the microfiltration range (typically, from 0.1 to 0.5 μm). The pure water is transferred as vapor from the feed to the permeate side and, depending on the MD configuration, can be recovered as a liquid inside or outside the module. Specifically, three main configurations are applied (see Figure 4): (i) direct contact membrane distillation (DCMD); (ii) air gap membrane distillation (AGMD); (iii) vacuum membrane distillation (VMD). In DCMD and AGMD, the vapor condenses inside the module: directly into the distillate stream at the permeate side (DCMD) or onto a cold surface of the module (AGMD). In both cases, thermal energy is needed to heat the water feed and to cool the distillate/cold stream. In VMD, the vapor is condensed outside the module and thermal energy is needed to heat the water feed and to transform the vapor into liquid in the condenser. In addition to the thermal energy, the electric energy for the recirculation of the streams and, in VMD, for the vacuum pump, must be considered, even if it usually has a much lower impact than thermal energy consumption. Modifications of the AGMD configuration, the so-called permeate gap membrane distillation (PGMD) and liquid gap membrane distillation (LGMD), were also analyzed (Figure 4d). In PGMD, the condensed permeate is extracted from the top of the module, so that the gap between the membrane and the cold surface is filled with permeate during the operation. In LGMD, the air gap is directly filled with liquid (water). In both configurations, the liquid layer into the gap absorbs both the latent heat of the vapor condensation and the sensible heat conducted through the membrane and transfers it to the cooling liquid. This translates into a lower mass transfer resistance and heat loss in the gap than those registered in the AGMD configuration. Another MD configuration investigated, though to a much lesser extent, is sweep gas membrane distillation (SGMD), based on the use of a sweep gas at the permeate side to strip the vapor, which is separated from the gas in a condenser outside the module.

## 3. Case Study: Desalination by RO and MD

**Figure 5:**
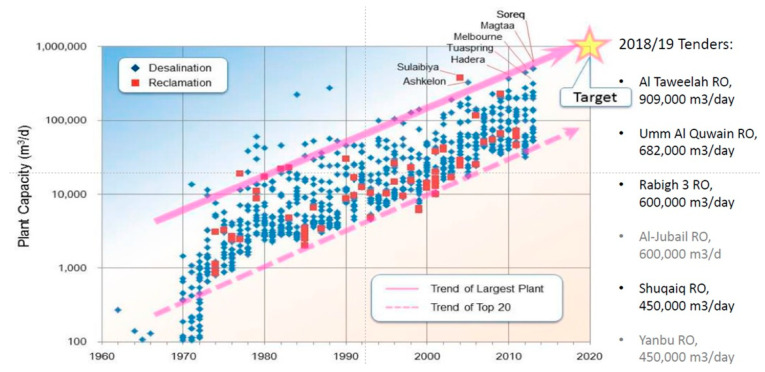
Trend of RO desalination plant capacity. Reproduced from [7] with Open Access.

The exacerbation of water stress made desalination a key solution to meet freshwater needs. Thermal desalination technologies, such as multi-stage flash distillation (MSF) and multi-effect distillation (MED), were the first to be employed. Typical energy consumption ranges from 10 to 15 kWh/m^3^ [5], with low freshwater recovery (20–40%) and high footprint. The development of high-selective and permeable membranes opened up the possibility of the application of RO in desalination. RO units operate at ambient temperature, are more compact, and can produce up to 50% freshwater recovery. Another membrane-based technology tested in desalination is electrodialysis (ED), which uses ion exchange membranes under an electrical field to separate salt from the seawater. However, ED was found to only be economically advantageous up to feed salinities of 3 g/L, confirming RO as a good choice for seawater desalination [6]. The number of desalination plants is constantly increasing, with RO currently representing the dominant technology. The capacity of RO plants has, in fact, significantly increased, moving from 1000 m^3^/d in 70 s up to 1,000,000 m^3^/d in 2020 (Figure 5).

**Figure 6:**
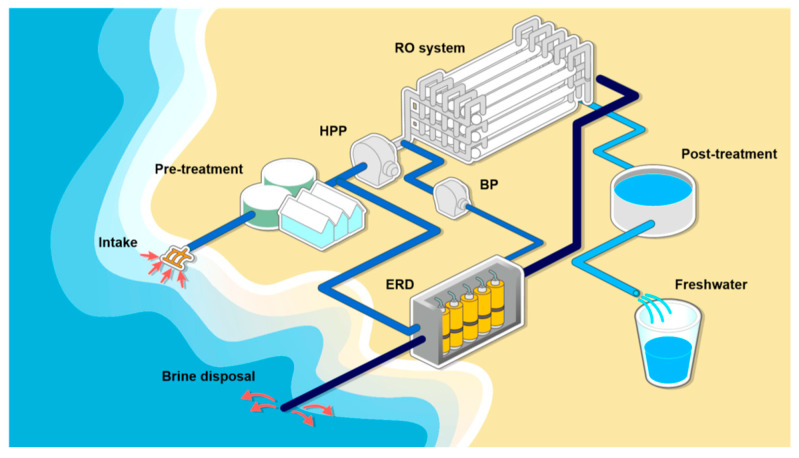
Energy Recovery Devices (ERDs) in a SWRO desalination plant. HPP—high pressure pump; BP—boosting pump. Reproduced from [8] with the permission from the Publisher. Copyright 2019, Elsevier.

In desalination by RO, electric energy must be supplied to achieve the separation of salt from seawater. The RO unit is fed pressurized seawater and produces two streams: the fresh water, which is at atmospheric pressure, and the brine, at high pressure. The seawater reverse osmosis (SWRO) specific energy consumption (SEC) of the first desalination plants was >6 kWh/m^3^. The design and optimization of energy recovery devices (ERDs) to recover pressure from the brine allowed the SEC to be reduced to 3–4 kWh/m^3^, which is the actual value when including both pre- and post-treatments. In this case, as it is shown in Figure 6, before it reaches the high-pressure pumps (HPP), part of the pretreated seawater is sent to the ERD, where it recovers pressure from the brine, before being fed to the RO system.

**Figure 7:**
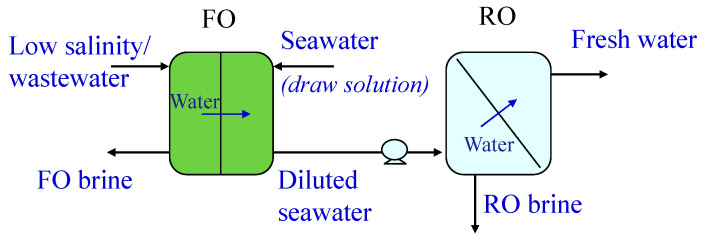
Forward osmosis (FO) as a pre-treatment to reduce the salinity of the RO feed. The seawater is first diluted in the FO unit and then sent to the RO for freshwater production.

**Figure 8:**
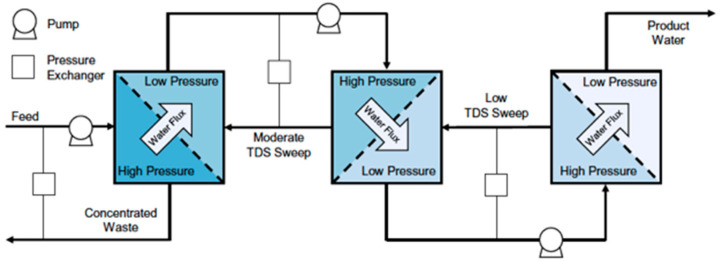
Osmotic-assisted RO (OARO) for high-salinity feeds. The feed is sent to two stages in series before the RO unit. Reproduced from [12] with the permission from the Publisher. Copyright 2017, Elsevier.

Another approach investigated to reduce the SEC in SWRO involves the dilution of seawater before it enters the RO units, since by lowering the feed concentration, the pressure needed for permeation is also lowered. In particular, two systems were studied and compared as a pre-treatment: forward osmosis (FO) [9,10,11] and osmotic-assisted RO (OARO) [12,13,14]. In the FO-RO system, seawater is used as a draw solution to concentrate a low salinity/wastewater stream so that a diluted seawater can be sent to the RO unit (Figure 7). The low salinity/wastewater (feed) is sent to one side of the FO membrane, while the seawater (draw solution) flows at the other side. The unit works at atmospheric pressure. Due to the difference in osmotic pressures between the feed and the draw solution, water moves through the membrane from the feed, which is concentrated (FO brine), to the seawater, which is diluted. The diluted seawater is finally sent to the RO unit to recover fresh water at lower operating pressures (therefore at lower energy consumptions). In OARO, different stages of permeation are present before the RO unit, each one equipped with pressure exchangers as ERDs, working with a lower concentrated stream at the permeate side, in order to reduce the osmotic pressure difference. In this way, the concentration of the stream sent to RO is significantly lower than that of the original feed. This solution was proposed mainly for high salinity feeds. Figure 8 shows an OARO system with two stages before the RO unit. The high salinity feed is sent under pressure to the first stage at one side of the membrane, while at the other side it is sent a low-pressure saline stream (less concentrated than the feed) in order to establish a hydraulic pressure difference across the membrane that is higher than the osmotic pressure difference. The water moves from the feed to the permeate, which is diluted and is sent, after its pressurization, as feed to the second stage. In this stage, a low-pressure saline stream (less concentrated than the permeate produced in the first stage) is sent to the other side of the membrane to receive water from the feed. The diluted permeate stream produced in the second stage is finally pressurized and sent as feed to a “classical” RO module, from which freshwater can be recovered.

The two options were compared to RO in terms of SEC and capital cost and, as seen in Table 1, they both led to a reduction in SEC, but at a high capital cost.

**Figure 9:**
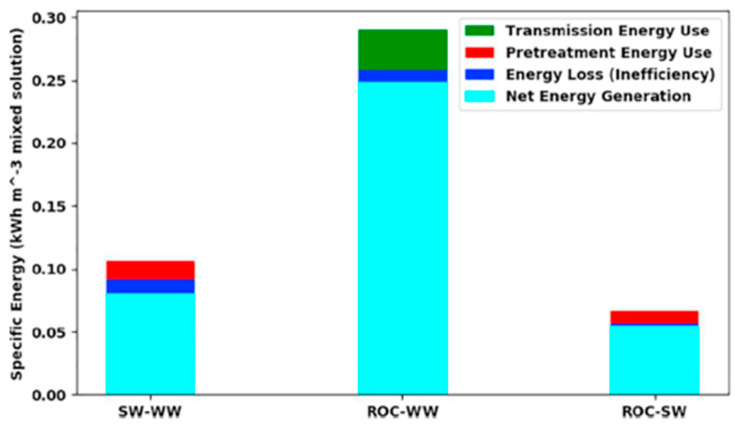
Energy involved in a Tampa Bay SWRO desalination plant by PRO working with different draw/feed streams. Reproduced from [17] with the permission from the publisher. Copyright 2019, Elsevier.

The pressurized RO brine was used to produce energy in pressure-retarded osmosis (PRO) units [16,17,18]. In PRO, the RO brine can be employed as the draw solution at the permeate side of the membrane, while a solution with a lower concentration is sent at the feed side. The water permeating from the feed to the draw side leads to a dilution of the pressurized stream, which can be used for power generation in a turbine. Benjamin et al. [17] analyzed the energy that can be generated in a Tampa Bay SWRO desalination plant by PRO working with different draw/feed streams. As shown in Figure 9, the highest net energy generation was obtained for the combination RO brine (ROC)/wastewater (WW), followed by SW/WW and ROC/SW. This result puts in evidence that if no low concentrated water (like WW) is available close by the desalination plant, a relatively low amount of energy can be produced with the only streams involved in RO (SW and RO brine).

**Figure 10:**
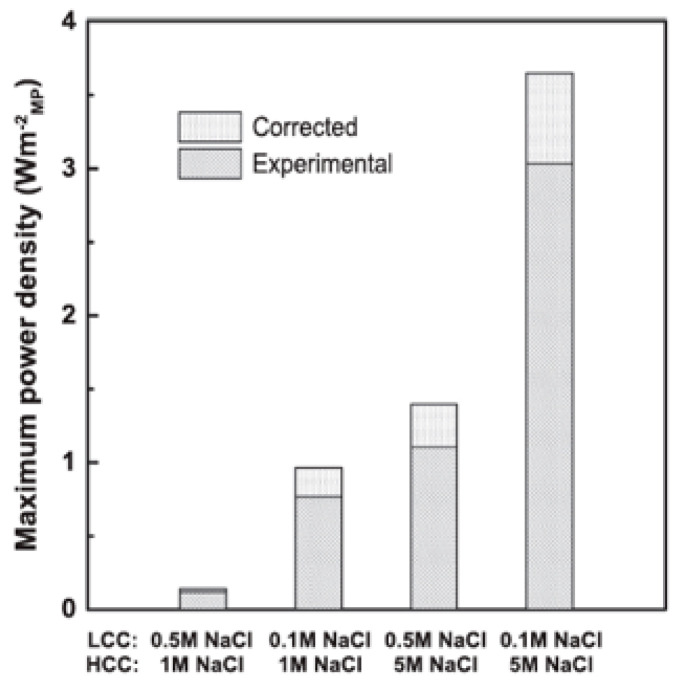
The maximum power density achievable by RED with different combinations of LCC and HCC streams. Reproduced from [19] with the permission from the Publisher. Copyright 2016, Elsevier.

The RO brine can also be used in reverse electrodialysis (RED) to create a salinity gradient power across ion-exchange membranes, through which anions and cations of the solution migrate, finally leading to electric energy production [19,20,21]. The efficiency of RED was studied by Tufa et al. [19] for different combinations of low-concentrated (LCC) and high-concentrated (HCC) feeds. In particular, two concentrations were considered for the LCC stream (0.1 M NaCl, representing brackish water, and 0.5 M NaCl, representing seawater) and the HCC stream (1 M NaCl, representing SWRO brine, and 5 M NaCl, representing a high-concentrated brine). The best results were registered when using the high-concentrated brine (see Figure 10). The highest maximum power density was obtained when operating at the highest difference in concentration (brackish water and high-concentrated brine), while the lowest was obtained for seawater/RO brine. An increase in the maximum power density, although always lower than the values obtained with the high-concentrated brine, was achieved when the RO brine was coupled with the brackish water. Therefore, if no low-concentrated water is available close by the desalination plant, nor the RO brine can be further concentrated, the energy that can be produced with the only streams involved in RO (SW and RO brine) is not viable. Figure 10 is reproduced from [19] and shows both experimental and corrected values, extrapolated by authors for the case of a stack with negligible effect of the resistance of the electrode compartments, as in the case of a high number of cell pairs.

The use of renewable energies in desalination represents a strategy adopted in recent years to reduce fossil fuel consumption in energy production. In particular, among the different renewable energies, solar energy was the most used one [22,23]. Solar-powered RO desalination plants were tested by different groups. Although efficient, the solar system suffers from variable power during the day and high footprint. To overcome these issues, the combination with the grid or batteries, to have a constant energy supply, and the use of solar concentrators, to reduce the footprint, are under investigation.

**Figure 11:**
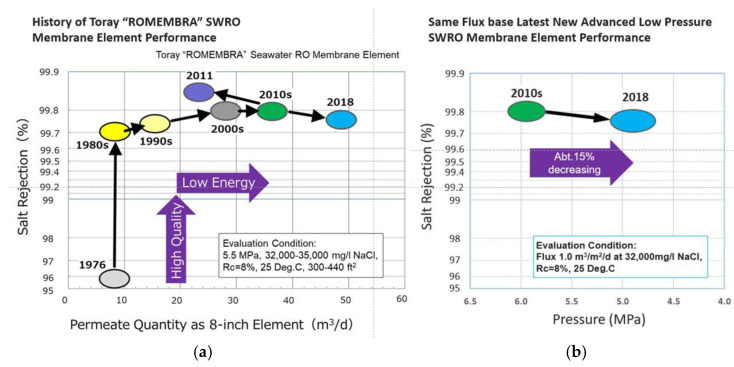
Improvements in SWRO membranes. (**a**) Trend of salt rejection from 1976 to 2018; (**b**) performance of the new advanced low pressure SWRO membrane. Reproduced from [7] with Open Access.

In addition to the approaches described, efforts were also made to act on the performance of SWRO membranes. As an example, Figure 11 shows the improvements made over the years by Toray. The first years focused on the development of membranes with higher separation efficiency (from 96% salt rejection in 1976 to 99.7% salt rejection in 1980s). Afterwards, research addressed the reduction in the energy consumption. From 2010 to 2018, a 15% decrease in the operating pressure was achieved, at parity of water flux and salt rejection, thanks to their so-called “new advanced low pressure SWRO membranes”.

The production of innovative high-flux SWRO membranes to facilitate water transport by reducing the membrane resistance and, then, the operating pressure, was also investigated by different researchers [24,25]. Three main membrane types were produced: (i) biomimetic [26,27]; (ii) with nanotubes [28,29]; (iii) nanocomposites [30,31]. Biomimetic membranes are based on the use of aquaporins and are a hundred times more permeable than conventional SWRO membranes. However, no data on their energy consumption are available yet. Only modeling results are currently available for membranes based on nanotubes, for which a ten times higher flux and a 30–50% lower energy consumption than available membranes were calculated. Nanocomposite membranes contain zeolite nanoparticles in the polymeric matrix and resulted in a more than doubled flux and a 20% lower energy consumption than conventional SWRO membranes. Despite these positive findings, information on the stability of the three types of membranes in long-term runs, and under high operating pressure, is still missing.

**Figure 12:**
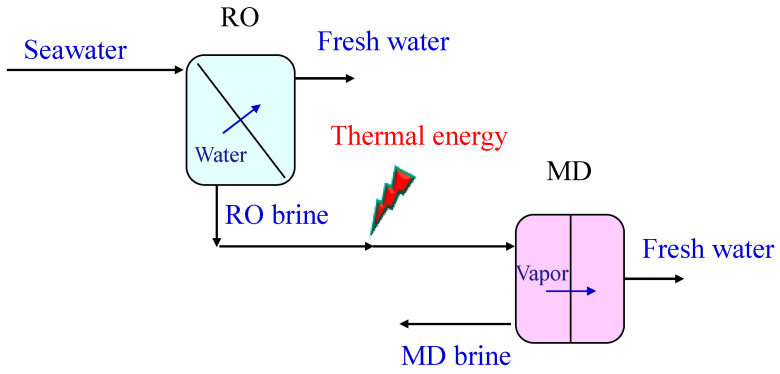
Scheme of an RO–MD integrated system in desalination. The RO brine is sent as feed to the MD unit where it evaporates, producing more fresh water.

The brine produced in RO is often discharged back into the sea or collected into solar ponds. However, this practice is going to change in the future because of its environmental impact [32,33,34]. In this respect, membrane distillation does not suffer from osmotic limitations, like RO, and can be used to further exploit the RO brine to increase the freshwater recovery, while reducing the brine to be disposed. Figure 12 shows the integration of an MD unit with the RO one, where the RO brine is sent to the MD unit as feed for further concentration. In this way, more freshwater is extracted from the RO brine, as distillate. The RO-MD integrated system overcomes limits of single units, leading to an improvement in the overall performance. In particular, with respect to the single RO unit, the presence of the MD leads to an increase not only in freshwater production, but also in its quality, due the 100% MD rejection for all non-volatiles contained into the water, including boron or trivalent arsenic, for which RO has a limited rejection. Moreover, with MD, the brine volumes to be disposed can be reduced and, if pushing MD up to feed oversaturation, salt crystals can be obtained from the brine, approaching the zero-liquid-discharge target. On the other hand, MD typically has lower fluxes than RO, and its combination with RO, rather than its use as standalone unit in desalination, allows the decrease of the MD membrane area needed at parity of operating conditions and the final productivity target. Furthermore, RO brine can reach a temperature up to 35 °C. Therefore, the MD feed is already pre-heated in the RO-MD integrated unit, with respect to the case in which MD is applied to treat directly seawater. Nevertheless, also in the RO-MD system, the RO brine needs to be warmed up to desired temperature before entering the MD module; thus, thermal energy must be supplied. To reduce the thermal demand, different types of heat recovery were proposed, depending on the MD configuration, as summarized hereinafter.

**Figure 13:**
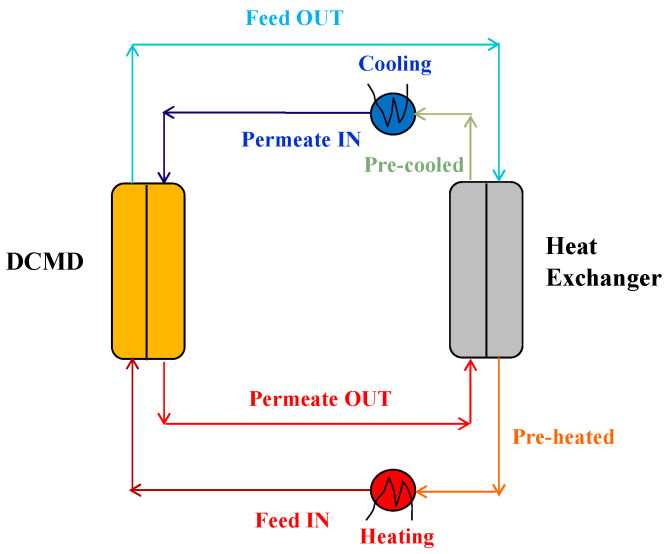
Pre-heating and pre-cooling of DCMD streams in a heat exchanger (HX) for heat recovery.

In DCMD, the heat of condensation acquired by the permeate stream can be used to pre-heat the feed exiting the module before its recirculation, as sketched in Figure 13. In this way, the permeate is also pre-cooled before being recycled back to the DCMD module.

**Figure 14:**
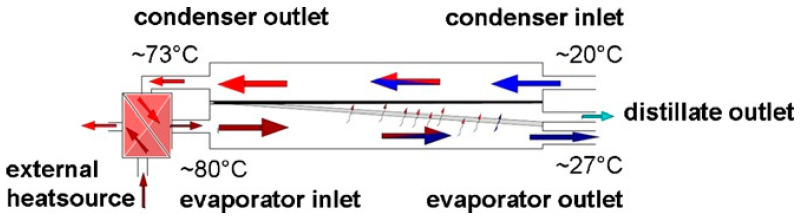
Heat recovery inside the module in AGMD. Reproduced from [35] with the permission from the Publisher. Copyright 2011, Elsevier.

In AGMD, heat recovery can be achieved directly inside the module by using the feed as the coolant stream of the condensing surface [35,36]. In Figure 14, the feed enters at 20 °C and becomes warmer (up to 73 °C) inside the module, thanks to the heat of condensation of the vapor on the cold surface. After, it is heated up to 80 °C in an external heat exchanger before entering the module again in the evaporator channel.

**Figure 15:**
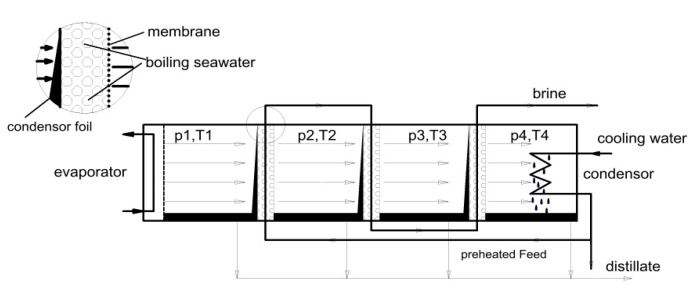
The memsys design. A series of effects with membranes and condensing foils is used for continuous evaporation and condensation. Reproduced from [38] with the permission from the Publisher. Copyright 2012, Elsevier.

A vacuum multi-effect membrane distillate (V-MEMD) technology (Figure 15) combining the advantages of multi-effects and vacuum was also designed by memsys [37] to obtain a high-efficiency heat recovery system [38]. The memsys module consists of a series of stages (each one called effect) with membranes and condensing foils for continuous evaporation and condensation. A steam riser is used to generate steam in the first effect, which is condensed at the surface of the condensing foil contacting, at the other side, the colder feed. In this way, the feed receives the latent heat of condensation through the condensing foil and evaporates through the membrane. The produced vapor is condensed, in a similar way, in the second effect. The feed evaporation and vapor condensation continue in all effects. The vapor generated in the last effect is condensed in a condenser. In particular, as reported in Figure 15, the feed can be used in the condenser as a cold liquid to be pre-heated before entering the first effect. The feed exiting the last effect is highly concentrated (brine).

Despite the different heat recovery strategies, actual MD pilot units to treat 70 g/L RO brine need a specific thermal energy consumption (STEC) of around 300 kWh/m^3^, which is still quite a high demand. However, MD is characterized by the use of lower operating temperatures than traditional distillation columns, and therefore, several “free” energy sources were explored, like solar [39,40,41,42,43], geothermal [44], waste low-grade heat [45], and salt gradient solar ponds (SGSPs) [46,47]. Among them, solar energy and SGSPs were the most investigated methods for desalination. Solar-powered MD systems were developed with different geometries of solar collectors: flat plate, evacuated tube, and compound parabolic [48,49,50,51,52,53]. Solar collectors were connected with MD units in a single loop or two-loop configurations [49,54]. The single-loop solar MD system includes a solar collector, often flat plate, and an MD module. The feed water is sent first to the solar collector for heating and, after, to the MD module. The two-loop solar MD system has two separate loops (solar and MD), connected through a heat exchanger. Water is heated in the solar loop and used in the heat exchanger to warm up the salty solution to be treated in the MD module. The system includes also a thermal storage tank where the water is stored. This type of configuration was usually proposed for large-scale desalination plants. In some studies, photovoltaic panels (PV) were also integrated into the solar-powered MD system to transform the thermal energy into the electric energy needed to run pumps [49,55].

**Figure 16:**
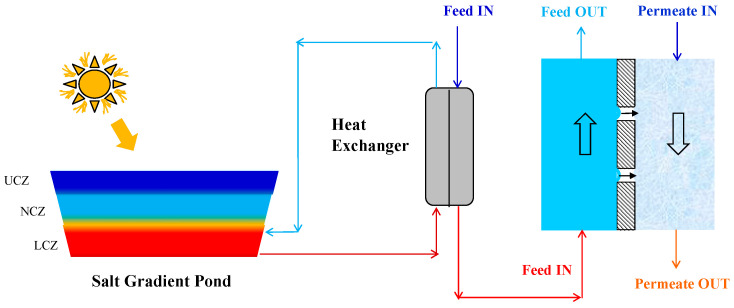
SGSP to heat the feed of a DCMD unit. The hot brine of the LCZ is sent to a heat exchanger to heat the feed to be treated by DCMD.

To improve the performance of solar MD systems in desalination, in addition to the different MD modules previously described, other MD module modifications were carried out, as summarized hereinafter. A finned tube AGMD module was developed by Cheng et al. [56], in which a finned copper tube, acting as support, was covered by a hydrophobic membrane. The feed flowed outside the tube, while the cold liquid was sent into its lumen. The gap between the membrane and the tube was the condensing chamber. A module containing ten finned tubes was designed and tested for solar-driven desalination, leading to an average flux of 17 kg/m^2^h, higher than that achieved with “traditional” AGMD modules reported in the literature. To reduce maintenance and moving parts, as often needed in remote and small-scale desalination plants, immediate assisted solar air gap membrane distillation (IAS-AGMD) was proposed by Summers and Lienhard [57]. In the system, the feed is directly heated at the membrane surface through an absorber plate. The plate absorbs the solar radiation and transfers heat to the feed. In this way, the solar-driven MD system is more compact, and the feed is more uniformly heated. Ho et al. [58] reported a 13.6% higher water production than the conventional AGMD. However, this system cannot be easily extended to large-scale applications, because the solar energy collected is limited by the radiation received per unit area. Another approach to reduce complexity and size of solar-driven MD plants was to combine an evacuated tube solar collector and a membrane distillation unit [41]. In particular, the membrane distillation modules were built directly into the evacuated solar tubes. In this system, the feed enters the evacuated tubes where hollow fiber membranes (0.2–0.4 μm pore size) are located. When the feed is heated, vapor is transferred through the membrane from the feed to the permeate side and it is condensed outside the evacuated tube in an external heat exchanger. A vacuum pump at the condensate side enhances the driving force (vacuum ∼10 kPa). The proposed system led to 4 L per day production of freshwater when a 1.6 m^2^ solar absorbing area was coupled with 0.2 m^2^ MD membranes, resulting in an interesting option for the rooftops of residential and commercial buildings. The variety of operating conditions, sizes, and solar systems reported in the literature studies does not allow a direct comparison of the performance of the various MD module designs. Nevertheless, some data can be found for commercial MD modules tested in the same conditions. For instance, Zaragoza et al. [48] compared four commercially available MD modules. The V-MEMD configuration emerged as the one with a low STEC (185 kWh/m^3^−380 kWh/m^3^) and a concentration factor one order of magnitude larger than in the single-effect technologies. However, authors pointed out how this configuration needs more electric energy for the vacuum system. Besides the development of innovative MD modules, multi-stage MD configurations were also investigated as a strategy to increase the performance. In this case, several identical MD modules were connected in series, parallel, or in series and parallel. Kim et al. [39] evaluated the performance of a 24-stage solar-powered VMD system, where ten external heat recovery units (HRU) were used to recover the latent heat of condensation. The VMD unit was based on commercial shell-and-tube capillary membrane module equipped with an array of polypropylene hydrophobic fibers. Shell-and-tube was also the configuration of the HRU. By increasing the number of HRUs, the system performed better in terms of both productivity and STEC. For instance, a 34% higher productivity and a 20% lower STEC were obtained with respect to the use of a single HRU. Guillén-Burrieza et al. [59] evaluated the performance of a solar-powered multi-stage AGMD and found a water recovery ratio of a three-stage AGMD 2.24-fold higher than that of a single stage. Guillén-Burrieza et al. [60] used commercial AGMD modules for two configurations: 1. six modules connected in parallel; 2. modules divided into two parallel connections with three AGMD modules in series connection. The second configuration performed better both in terms of distillate production and heat recovery, with a PR (ratio of the mass of distillate produced to the heat input) of 1.96 versus 0.53. Based on the above results, it seems clear that the multi-stage MD system with heat recovery could be a good option for improving the overall performance of solar-driven MD desalination. Another source of “free” thermal energy often investigated for MD in desalination is that supplied by SGSPs. SGSPs are saline-stratified water ponds used as both a heat source and as heat storage. SGSPs consist of three main layers: the top layer (upper convective zone, UCZ), which is at atmospheric temperature and has a low salt concentration; the intermediate layer (non-convective zone, NCZ), characterized by a salinity gradient and a temperature gradient; the bottom layer (lower convective zone, LCZ), at higher temperature and salt concentration. In Figure 16, the hot stream coming from the bottom of the water pond is used to warm up to the desired temperature the feed before a DCMD unit. Typical temperatures of this zone range between 50 and 90 °C. For instance, by using a 50 m^2^ SGSP, it was possible to treat a 1.3% salinity feed with DCMD, obtaining flux values ranging from 2.0 to 5.8 kg/m^2^ h when working at 30 °C and 45 °C, respectively [46]. Walton et al. [61] used the thermal energy of a SGSP to warm salty solutions of various concentrations (0.6–4 M NaCl solutions) for their treatment in a commercial AGMD module, achieving fluxes up to 6 L/m^2^h.

However, it is worth mentioning that SGSPs are suitable only for specific locations on Earth: near the ocean and with enough solar radiation. Suárez and Urtubia [47] reported locations between 40° N and 40° S as being suitable, for which the useful heat varied from 40 to 112 W/m^2^ with a water production ranging from 1.05 to 2.95 L/day m^2^ of solar pond, respectively. Interestingly, the area of membrane distillation units was always less than 1% of the solar pond area, an indication of the high footprint of SGSPs. Moreover, in their work, five selected sites were indicated: Cape Town (South Africa), Jeddah (Saudi Arabia), Darwin (Australia), Los Angeles (USA), and Copiapò (Chile). A comparison between solar systems and SGSPs was carried out by Méricq et al. [62] who studied two configurations for both SGSPs and solar collectors (SCs). In one configuration, the hot feed was sent to the MD module, while in the other configuration the membrane module was directly heated by submerging it in the SGSP or by integrating the SC onto the module-self, at the feed side. VMD was the investigated MD configuration in a wide range of operating conditions (0–300 g/L of feed concentration; 100–10,000 Pa of permeate pressure; 20–70 °C of feed temperature). The submerged module in the SGSP, although it represented the solution with the lowest energy requirement, was characterized by the lowest fluxes due to a significant temperature and concentration polarization as well as high maintenance and difficult technical feasibility. By coupling the MD module with the SGSP, higher fluxes were obtained, but still considerable maintenance was required. Both configurations with SCs led to higher flux values, required little maintenance, but resulted in being more expensive than SGSPs. The integration of the SC onto the module-self was more difficult from a technical point of view and could lead to temperature radial profiles, with a consequent reduction in flux, with respect to the use of SC to heat the feed outside the module. Therefore, the authors concluded that the use of SC, with the hot feed sent to the module, should be the preferred option.

**Figure 17:**
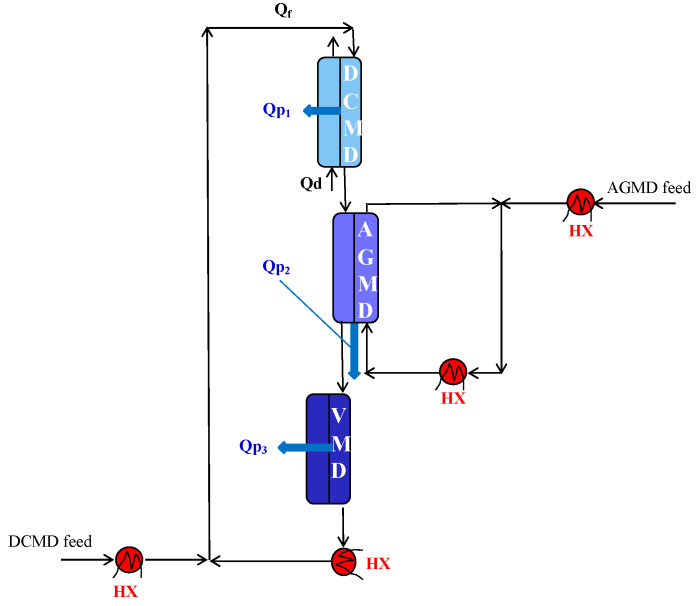
Integrated DCMD-AGMD-VMD system. The feed exiting DCMD is first used as cold stream in AGMD, where it receives the heat of condensation of the vapor produced in AGMD, and after is further treated as feed in VMD.

Other strategies to reduce the thermal energy consumption in membrane distillation were investigated, even if only currently at a research stage/small scale. For example, in pressure-retarded membrane distillation (PRMD), the volume increment of the condensation liquid was used to drive a hydraulic turbine. This system allowed 188 L of high-quality freshwater to be produced from 0.5 M NaCl solution with 27.8 kJ power per day for a 1.0 m^2^ heating area [63] and can be applied also for the treatment of more concentrated streams, like RO brines. The integration of different MD configurations was also proposed as a possible way to reduce the STEC in membrane distillation [64,65,66]. This strategy was developed to cover also the applications where low feed temperatures (e.g., 40 °C) are needed, and for which heat recovery systems cannot be applied. In particular, DCMD was coupled with both AGMD and VMD units. AGMD replaced the conventional heat exchanger to supply the heat lost by the feed stream in the DCMD unit through the heat of vapor condensation, while producing more permeate (Qp). VMD was considered to improve the overall permeate production. Various combinations were investigated. In the DCMD-AGMD-VMD integration (Figure 17), the stream exiting DCMD was sent as a cold liquid in AGMD, where it was heated by receiving the heat of condensation of the vapor produced in AGMD. Afterwards, the heated stream was sent to a VMD unit where it was further treated to produce more permeate. In Figure 17, the HXs needed to warm up the involved streams up to the desired temperatures are also shown. When compared to the DCMD-HX conventional system, the DCMD-AGMD-VMD integrated configuration led to a 515% higher permeate production and a 48% lower STEC [66]. Moreover, the MD integrated units resulted to better fit the logic of the process intensification, showing a productivity/size ratio [67] greater than that of the conventional system.

A phenomenon affecting MD performance is the temperature polarization, which consists of the creation of a temperature profile close to the membrane surface so that the membrane surface temperature is lower than that of the bulk feed. The lower the surface temperature, the lower the trans-membrane flux across the membrane, and therefore, the lower the permeate production. To face this issue, specific module designs and spacers to promote the turbulence at the feed side were realized and tested [68,69]. More recently, membrane modifications were investigated such as the preparation of more porous structures, to reduce the heat loss through membrane material and photo-thermal membranes and electro-thermal membranes, to increase the temperature at the membrane surface thanks to solar and electric heating, respectively [70,71,72,73,74,75,76]. In both cases, the temperature at the membrane surface can reach values higher than the bulk feed temperature (a sort of “reverse temperature polarization”), with consequent improvements in permeate production and reduction in energy consumption. Photothermal membranes were prepared by including photothermal nanoparticles (often metals (e.g., Ag) or carbon-based nanomaterials) in the polymeric structure or by coating the membrane surface with them. Modules with quartz or a transparent (polymethylmethacrylate (Plexiglas)) window were also realized to allow the irradiation of the nanoparticles. Once irradiated, nanoparticles converted the solar energy into thermal energy, which allowed the water evaporation. Electro-thermal membranes were mainly obtained by coating the membrane surface with layers of carbon nanotube. Afterwards, the prepared membranes were connected to an electric circuit through which a current was supplied in order to heat the fluid via Joule heating (conversion of electric energy into heat energy when an electric current passes through a conductive material). For both photothermal and electro-thermal membranes, the nanoparticle concentration represented one of the key parameters for performance optimization. In the literature, the use of both types of modified membranes led to an improvement of flux with respect to the pristine membranes tested in the same conditions. Specific energy consumptions (defined in this case as the amount of total energy supplied (thermal and electric) with respect to the produced distillate) ranged from 0.22 to 2.3 kW/kg and from 0.11 to 11.86 kW/kg for the photo-thermal and electro-thermal membranes, respectively [77]. The high variability registered in SEC values for both modified membranes depends on many factors, like the type and amount of nanoparticles used, the final membrane properties obtained (pore size, thickness, porosity, etc.), the MD configuration investigated, the feed concentration and flowrate, the module design (including the fluid-dynamic developed inside the module), and the amount of energy supplied. This is because the research in the field is still in progress and different aspects are under investigation. Specifically, the smallest SEC of 0.11 kW/kg was obtained by Subrahmanya et al. [73] with a graphene–PVDF flat membrane Joule heater tested for VMD desalination in a module of 778.54 mm^2^ membrane area. At a feed flowrate of 1 mL/min, and with an energy supply of 2 W, a temperature of 56 °C was registered on the membrane surface, with a vapor flux of 23.44 kg/m^2^h.

**Figure 18:**
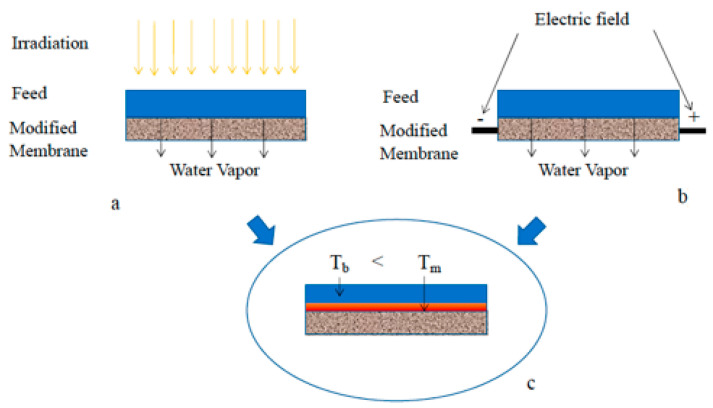
(**a**) Photo-thermal membrane under irradiation; (**b**) electro-thermal membrane under electric field; (**c**) temperature profile developed between the bulk feed and the membrane surface. Reproduced from [77] with Open Access.

Figure 18 reports the schemes of how the two types of modified membranes work.

**Figure 19:**
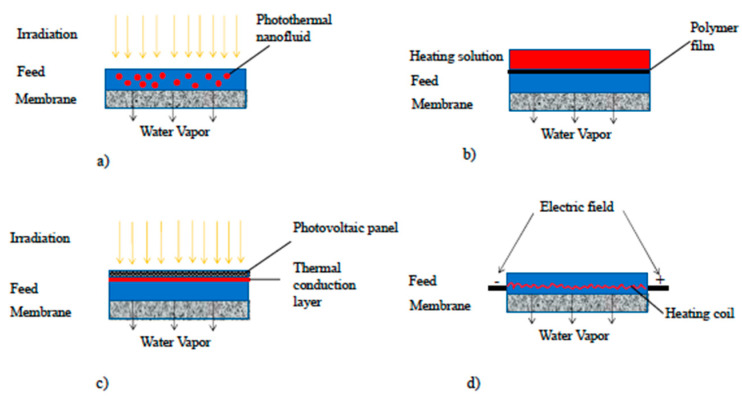

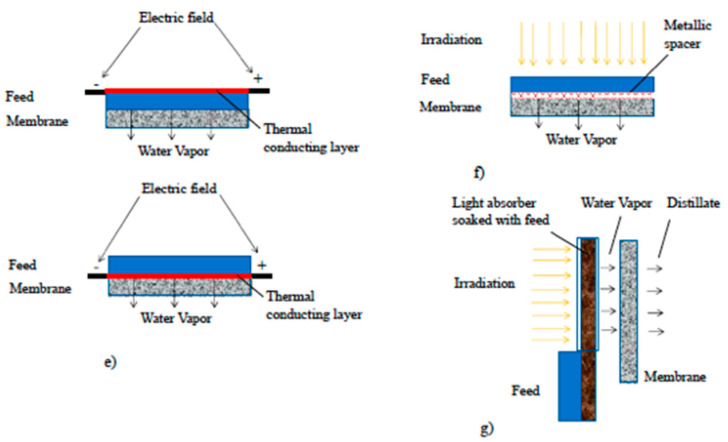
Localized heating inside modules. (**a**) Use of photothermal nanofluid inside the feed. (**b**) Heating of the feed through a polymer film in contact with an external heating solution. (**c**) Heating of the feed on the back of a photovoltaic panel through a thermal conduction layer. (**d**) Use of a heating coil inside the module at the feed side. (**e**) Heating of the feed through a thermal conducting layer connected to electricity. (**f**) Use of a metallic spacer inside the module at the feed side, close to the membrane surface. (**g**) Use of membrane distillation as a final purification step of the water vapor produced in a light absorber under irradiation. Reproduced from [77] with Open Access.

Both photo- and electro-thermal membranes need the modified membranes to be prepared first. Afterwards, in the case of electro-thermal membranes, the electricity has to be supplied, while for the photo-thermal membranes, a specific module to allow the irradiation to reach the membrane surface must be designed before the irradiation step. Localized heating can also be achieved inside the modules (without modifying the membrane), as shown in Figure 19 [78,79,80,81,82]. In particular, a possible strategy could be to use photothermal nanofluids in the feed, which is irradiated through a quartz window, so that nanofluids could absorb the solar radiation and transform it into heat (Figure 19a). The feed can also be heated through a polymer film in contact with a heating solution (Figure 19b) or by applying a thermal conductive layer on the backside of a photovoltaic panel (Figure 19c). In the latter, the waste heat of the solar cell can be transferred through the layer to the feed, which is heated. A heating coil inside the module, located close to the membrane surface and connected to an electric circuit, can be another way to heat the feed inside the module (Figure 19d). Similarly, thermal conductive layers connected to electricity can be placed inside the feed channel, also close to the membrane surface, for the feed heating (Figure 19e). The use of metallic spacers coated with photocatalysts and located close to the membrane was also studied as an option for heating the feed under irradiation (Figure 19f). An alternative system was investigated by Gong et al. [83] to reduce fouling issues of the MD membrane. In this system, the membrane was not in direct contact with the feed, which is absorbed by a graphene–nickel foam with a polymer coating. Under irradiation, the feed evaporated. The produced vapor, after migration into a gap, moved through the membrane, which blocked all microorganisms at one side and led to the final distillate at the other side (Figure 19g). All the systems investigated led to an improvement of flux and to a reduction in energy consumption, with a SEC ranging from 0.58 to 2.8 kW/kg [48]. As previously reported, also in this case, the high variability registered in SEC values depends on the different system designs and operating conditions adopted.

**Figure 20:**
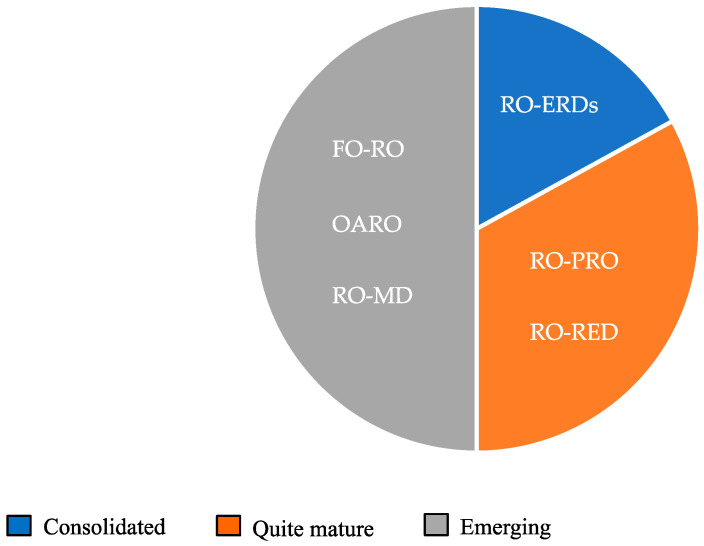
Degree of development of the different integrated RO systems.

Figure 20 summarizes the degree of development of the different integrated RO systems reported and discussed in this work. It can be noted that only the use of ERDs to recover the pressure from the RO brine is a consolidated system, while further optimization/development is required for the other combinations, especially for the FO-RO and RO-MD integrations, in order to apply them at a large scale.

## 4. Conclusions

The exacerbation of water stress, climate change, resource depletion, and environmental pollution need urgent action. Membrane operations are able to efficiently treat wastewater streams and produce freshwater from seawater, with membrane bioreactors representing the best available technology (BAT) for municipal wastewater purification and reverse osmosis becoming the dominant technology in desalination. However, in order to make membrane operations even more efficient from an energy point of view, moving towards the SDG 13 “Climate action”, different approaches were proposed and investigated, as reported in this contribution. In particular, in desalination, while the use of ERDs is today a well-established practice, the combination of RO with FO, PRO, and RED, as well as the OARO system, are linked to the availability of other (diluted) streams not always present in/close to desalination plants. Moreover, even if these combinations result as being effective in reducing the RO energy consumption, they lead to an increase in both plant complexity and capital/operating costs. Another option is the development of high-flux RO membranes (based on aquaporin, nanotubes or nanocomposites), which seem to be promising but need further investigation to be assessed in long-term runs. The coupling of RO to MD is also an interesting approach to improving freshwater production (SDG 6: Clean water and sanitation) and quality (SDG 6: Clean water and sanitation; SDG 3: Good health and well-being) and to reducing the RO brine that is disposed, reaching zero-liquid-discharge when applying MD to the formation of salt crystals (SGD 14: life below water). In this respect, various methods were analyzed to reduce the thermal energy demand of the MD unit. Among them were the optimization of MD modules with heat recovery and cascade design, the integration of different MD configurations, and the use of multi-stage MD configurations. Moreover, the latest trend of research focuses on the localized heating approach as a way to reduce the temperature polarization phenomena. In particular, both photo-thermal and electro-thermal heating proved effective in improving the MD efficiency. However, the research in the field is still in progress and different aspects are under investigation; thus, a high variability is registered in SEC values because of the different system designs and operating conditions analyzed. Therefore, future research should be address the optimization of the photo-thermal and electro-thermal heating systems, with a specific emphasis also on their sustainability, in order to avoid nanoparticles/materials leaching in the environment during long-run tests in real fields. In addition to the described research efforts, the use of different sources of “free” energy is another aspect investigated for both RO and MD. Solar-powered RO and MD plants seem to be the most promising one for areas with a high solar irradiance. However, the intermittent nature of solar radiation affects the productivity and the operational period of the solar systems, requiring storage units and grid lines, with an impact on the overall water production cost. To address this, the development of low-cost batteries with high storage capacities and the coupling of the solar powered units with a controller able to select the number of modules to use based on the solar radiation availability could be considered. From the above scenario, it is expected that the efforts in progress will contribute to a further consolidation of membrane operations in desalination as sustainable technologies for freshwater production at reduced energy impact.

## Figures and Tables

**Table 1:** Comparison between FO-RO, OARO, and SWRO based on data of [15].

Desalination Process	SEC (kWh/m^3^)	Normalized (to SWRO) Capital Cost
SWRO	2.5–4.0	1.00
FO-RO	1.3–1.5	1.21
OARO	1.8	1.15

## Data Availability

No new data were created in this review.
